# Diagnostic accuracy of glycogen phosphorylase BB for myocardial infarction: A systematic review and meta‐analysis

**DOI:** 10.1002/jcla.24368

**Published:** 2022-03-24

**Authors:** Anup Ghimire, Subarna Giri, Niharika Khanal, Shivani Rayamajhi, Anjila Thapa, Anil Bist, Surya Devkota

**Affiliations:** ^1^ Maharajgunj Medical Campus Institute of Medicine Tribhuvan University Kathmandu Nepal; ^2^ 567553 Department of Cardiology Manmohan Cardiothoracic Vascular and Transplant Center Kathmandu Nepal

**Keywords:** cardiac biomarker, diagnosis, glycogen phosphorylase BB, myocardial infarction

## Abstract

**Purpose:**

We tried to investigate the diagnostic accuracy of glycogen phosphorylase BB as a cardiac marker for myocardial infarction.

**Methods:**

We searched through different electronic databases (PubMed, Google‐scholar, Embase, and Cochrane Library) to locate relevant articles. Studies, with sufficient data to reconstruct a 2 × 2 contingency table, met our inclusion criteria were included. Three reviewers independently screened the articles. Discrepancies were resolved by other reviewers. Unpublished data were requested from the authors of the study via email. Subsequently, data extraction was done using a standardized form and quality assessment of studies using the QUADAS‐2 tool. Meta‐analysis was done using a bivariate model using R software.

**Results:**

Fourteen studies were selected for the final evaluation, which yielded the summary points: pooled sensitivity 87.77% (77.52%–93.72%, I^2^ = 86%), pooled specificity 88.45% (75.59%–94.99%, I^2^ = 88%), pooled DOR 49.37(14.53–167.72, I^2^ = 89%), and AUC of SROC was 0.923. The lambda value of the HSROC curve was 3.670. The Fagan plot showed that GPBB increases the pretest probability of myocardial infarction from 46% to 81% when positive, and it lowers the same probability to 12% when negative.

**Conclusion:**

With these results, we can conclude that GPBB has modest accuracy in screening myocardial infarction, but the limitations of the study warrant further high‐quality studies to confirm its usefulness in predicting myocardial infarction (MI).

## Graphical abstract

Our study is a systematic review and meta‐analysis done to evaluate GPBB as a potential biomarker for the prompt diagnosis of MI. 14 studies were selected for evaluation after a thorough screening. Our results (AUC = 0.923, DOR = 49.37) have led to the conclusion that GPBB serves as a moderately accurate biomarker for the early diagnosis of MI and could revolutionize the treatment protocol currently set for MI. We do recognize that our paper comes with its set of limitation therefore, further studies on the topic is still needed.

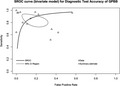

## 1 INTRODUCTION

Ischemic heart disease, also known as coronary heart disease (CHD) refers to a heart condition that is characterized by narrowing of arteries, which supply blood to the heart muscles. This narrowing can cause irreversible damage or death of the cardiac muscles due to severe deprivation of blood flow to a specific area of the heart, thus called myocardial infarction.^1^ Myocardial infarction is a pathological condition caused by disruption of the blood supply to a region of the heart to an extent that limits adequate oxygenation, even with an extended period of rest.^2^ According to the Global Burden Disease, the burden of IHD‐related deaths was 8.9 million in 2017, which is a 52.3% increase from 5.9 million in 1990,^3^ and acute myocardial infarction (AMI) is the leading cause of death among ischemic heart diseases.^4^ Most of the AMI‐related mortality occurs within the first hour of onset of symptoms.^5^ Hence, early detection of myocardial infarction (MI) is crucial to reduce coronary artery disease‐related morbidity and mortality.^6^

Since ECG alone has a low sensitivity and specificity for AMI diagnosis, the American College of Cardiology has included an elevation of cardiac troponin levels and other supplementary examinations in its guideline for AMI diagnosis.^7^, ^8^ Biomarkers are being used widely for the early detection of myocardial injury. To date, many cardiac markers have been identified for early identification of acute myocardial infarction,^9^ all varying in sensitivity and specificity. In current worldwise practice, the most commonly used biomarkers are cardiac troponins and creatine kinase (CK‐MB). Among the two isoforms of troponin, troponin T and troponin I, studies show that troponin I increases early, i.e., within 4–6 h, reaches peak concentration at 12 h, and finally returns to base level in 3 to 10 days.^9^, ^10^ Troponin T on the other hand remains elevated longer, i.e., 12–48 h, and falls to baseline level in about 10 days. Since cardiac troponins (Cantini) are found to have higher specificity and sensitivity over other cardiac enzymes, the elevation of cardiac troponin T is recommended as the standard biomarker criterion for establishing the diagnosis of acute myocardial infarction.^11^, ^12^

Similarly, another biomarker in practice is CK‐MB, which although present in a significant amount in heart muscle, is not heart‐specific as some of it is found in skeletal muscles and other tissues.^13^ CK‐MB rises early in the serum at almost 4–9 h after the onset of chest pain, reaches the peak concentration in blood nearly at ~24 h, and subsequently returns to baseline level at 48–72 h. Since CK‐MB is cleared from the circulation early, it has higher chances of detection of reinfarction over troponins.^14^ It has a specificity of 97 percent just 10–12 h after the onset of symptoms and good sensitivity if serial follow‐up is done for 24–48 h,^15^ but the diagnostic sensitivity is only 50% at 3 h.^16^

The search for biomarkers that could aid in the early detection of MI with high specificity and sensitivity has led to the discovery of many novel molecules. Among the many emerging novel biomarkers, glycogen phosphorylase BB (GPBB) stands out as it increases in the earliest hours after AMI has set. This hints at a prospect in the use of GPBB as an early marker of AMI before significant damage ensues.^5^ In most AMI patients, GPBB has been found to be increased as early as 1–4 h after the onset of chest pain and it usually peaks before CK‐MB or troponin T with subsequent return to baseline level within 1–2 days after AMI onset.^17^ These early results showing the potential use of GPBB for early diagnosis of AMI were our impetus in conducting this meta‐analysis to analyze its diagnostic accuracy in acute myocardial infarction.

## 2 METHOD

### 2.1 Protocol registration

The review protocol, with a comprehensive methodology, inclusion criteria, exclusion criteria, search strategy, and review questions, was registered in Prospero. Registration Number: CRD42021252095.

### 2.2 Information source and search strategy

We have analyzed the diagnostic accuracy of glycogen phosphorylase BB for myocardial infarction according to the Preferred Reporting Items for Systematic Reviews and Meta‐Analyses (PRISMA) guidelines.^18^ The following databases served as sources for published studies prior to March 2021 to locate relevant articles that address the review question: PubMed, PMC, Embase, Google Scholar, and Cochrane Library. Our comprehensive search strategy included the terms: “acute coronary syndrome”, “myocardial ischemia”, “coronary artery disease”, “glycogen phosphorylase, brain form”, and “glycogen phosphorylase” under the medical subject heading (MeSH) terms for MEDLINE, and relevant Emtree terms were used for EMBASE search. Boolean logic was used for conducting a database search, and Boolean search operators “AND” and “OR” were used to link search terms. Various grey literature libraries, preprint servers, and thesis repositories were searched for unpublished studies. Furthermore, a secondary search was done by screening the references of retrieved studies and previous systematic reviews. The applied search strategy can be accessed in the Appendix S1.

### 2.3 Inclusion and exclusion criteria

Eligible studies included in this meta‐analysis had to fulfill the following criteria:

1. Testing of GPBB in patients with suspected MI or having retrosternal chest pain,
2. Studies reporting sufficient data to reconstruct a diagnostic 2 × 2 table by a test of GPBB,
3. All patients diagnosed with standard methods for MI.

Articles were excluded for the following reasons:

1. Studies published in any language other than English,
2. Publications not related to the diagnostic value of GPBB or without enough data to reconstruct the diagnostic 2 × 2 tables,
3. Studies without valid data or with improper data and animal studies,
4. Reviews and case reports.

### 2.4 Data extraction

The references retrieved from different databases were imported into Covidence (a primary screening and data extraction tool). Covidence identified most of the duplicates and removed them automatically. The title and abstract of the remaining papers were then screened independently by NK, SR, and AT for potentially relevant studies that needed a full appraisal. AG, NK, and SR did a full‐text review of selected studies based on inclusion and exclusion criteria. Any discrepancies that arose were resolved by mutual discussion and consultation with other authors. Missing data were obtained via contacting the respective study author through email. Data were extracted from the shortlisted articles onto a standardized form designed in excel under the heading (author name, year, country, title, study design, exclusion and inclusion criteria, population characteristics, sensitivity, specificity, AUC value, cutoff value of GPBB used, and assay design used).

### 2.5 Assessment of methodological quality

All authors individually used the Quality Assessment Tool for Diagnostic Accuracy Studies (QUADAS‐2)^19^ to assess the methodological quality of the included studies.

Four domains were assessed for biases: (1) patient selection, (2) index test, (3) reference test, and (4) flow and timing.

### 2.6 Summary measures and meta‐analysis

We prepared a 2 × 2 contingency table for each of the studies with TP, TN, FP, and FN, which was then imported to R (R version 4.0.3 [2020‐10‐10]) for statistical analysis. The author AG did the statistical analysis. We adopted a random‐effect univariate statistical model using meta‐package to calculate the pooled sensitivity and specificity with metaprop function and diagnostic odds ratio with metabin function, with their corresponding 95% confidence intervals. The Clopper–Pearson method^20^ was used to calculate the confidence interval, and the Cochrane Q test^21^ and Higgins' I^2^ ^22^ were used to determine heterogeneity. Sensitivity analyses were performed by serially excluding each study to determine the outlier study and the effect of individual studies on the degree of heterogeneity using the leave‐one‐out method under metainf package. Representation of accuracy estimates from each study in a receiver‐operating characteristic (ROC) space and computation of Spearman correlation coefficient between the sensitivity and false‐positive rate were assessed for threshold effect. A typical pattern of the "shoulder arm" plot in a ROC space and a strong positive correlation would suggest a threshold effect. To address the nonthreshold heterogeneity, subgroup analysis and metaregression were performed considering several covariates. The SROC curve was constructed using a bivariate model (mada‐package; reitsma function) (sensitivity as the vertical axis, false‐positive rate as the horizontal axis), and the area under the SROC curve (AUC) was calculated.^23^ The hierarchical summary receiver‐operating characteristic (HSROC) was used to examine the GPBB accuracy for the diagnosis of MI. We evaluated pretest probabilities (as prevalence) versus corresponding post‐test probabilities following a positive or negative GPBB result based on the summary sensitivity and specificity using a Fagan plot. This showed the relationship between the prior probability, the likelihood ratio, and the post‐test probability.

### 2.7 Publication bias

The Deeks' funnel plot was used to assess the funnel plot asymmetry for publication bias.^24^

## 3 RESULT

### 3.1 Study selection

**FIGURE 1:**
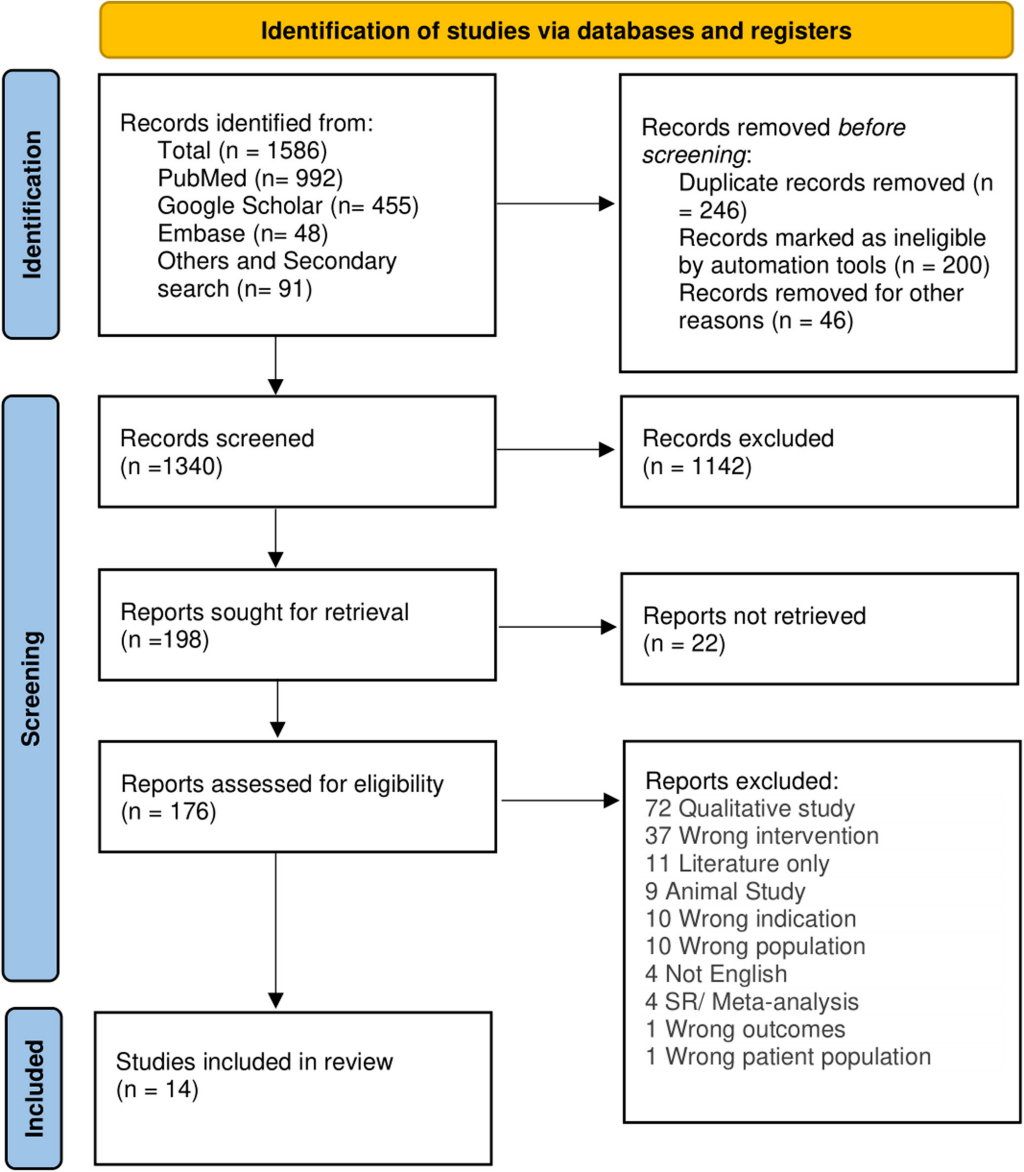
Flow chart illustrating the electronic database searches and selection of studies in the meta‐analysis

We retrieved a total of 1586 articles through our search strategies: 992 articles from PubMed and PubMed Central, 503 articles from Google Scholar and EMBASE, and 91 articles via secondary data search. Covidence removed 200 duplicate articles, and the remaining (n = 46) duplicates were removed manually, so 1340 studies were eligible for subsequent literature screening. We excluded 1142 irrelevant articles through title and abstract screening; 198 articles underwent full‐text screening using predefined inclusion and exclusion criteria. We excluded 184 articles among which 4 articles were systematic review/meta‐analysis and 72 articles that lacked quantitative analysis involving GPBB in MI. The remaining 14 articles were included in qualitative synthesis and meta‐analysis. The PRISMA flow diagram (see Figure 1) depicts our deployed study retrieval process.

### 3.2 Study characteristics

The total number of participants from all the included studies (n = 14) was 1680 with the number of cases and controls being 773 and 907, respectively. Included studies were from different geographical regions. Prospective (n = 5), case‐control (n = 4), and cross‐sectional (n = 4) were the predominant study designs used. All the patients had presented to their respective study centers within 6 h of symptoms onset. They were admitted to the emergency department or ICU or CCU of the same centers. Most studies used Diacordon as an assay design in measuring GPBB in the patient presenting with ACS‐like symptoms. The cutoff range of GPBB used in the included studies was between 6.5 and 19 ng/ml. All the studies except McCann et al. provided the data on the overall sensitivity and specificity of GPBB. The characteristics of individual studies are shown in Table 1.

**TABLE 1:** Table showing various characteristics (author, country, study design, inclusion criteria, total sample, cases, and control number, AUC, sensitivity, specificity, assay design, and cutoff) of various included studies

Author	Country	Study design	Inclusion criteria	Total population	Cases	Controls	AUC	Sensitivity (%)	Specificity (%)	Assay design	Cutoff value (ng/ml)
Vedika 2017^25^	India	Case‐control	Within 4 h of onset of chest pain	250	150	100	0.995	96	98	QAYEE‐BIO	7–18.47
Neelima 2017^5^	India	Case‐control	Within 4 h of onset of chest pain	200	100	100	0.993	96	99	ERBA ELISA kit	19
Ming 2017^26^	.	Prospective		155	72	83	0.845	77.8	89.2	Abbexa ELISA Kit	
Shortt 2013^27^	Canada	Letter to Editor	Within 6 h before presentation	163	11	152	0.67	67	58	Randox Evidence investigator	7.88
Zehra 2012^28^	Turkey	Prospective	Within 3 h after the onset of chest pain	117	45	72	0.93	93.2	78	Diacordon	14.3
Cubranic 2012^29^	Croatia	Cross‐sectional	CCU admission within 3 h of symptom onset	92	71	21	0.93	97	81	Diacordon	7
Bozkurt 2011^30^	Poland	Prospective	ED admission within an hour of symptom onset	72	24	48	0.82	95.8	43.7	Diacordon	10
Meune 2011^31^	.	Cross‐sectional	ICU admission within 6h after chest pain	60	31	29	0.55	50	64	Diacordon	10
Figiel 2011^32^	Poland	Cross‐sectional	Within 6 h from the symptom onset	52	50	2		64	100	Diacordon	
McCann 2008^33^	Ireland, UK	Prospective	Mean ED admission <4 h after chest pain	156	78	78	0.63	64		Diacordon	7
Mion 2007^34^	.	Cross‐sectional	Mean ED admission 3.8 h after chest pain	132	42	90	0.67	69	64	Randox Evidence investigator	6.5
Stejskal 2007^35^	Czech Republic	Case‐control	Mean ED admission 3.2 h after onset of chest pain	40	20	20	1	100	100	Diacordon	10
Peetz 2005^36^	.	Prospective	ED admission <2 h after chest pain	84	34	50	0.99	97	96	Diacordon	8.9
G Rabitzsch 1995^37^	Poland	Case‐control	Mean ED admission 4.7 h after chest pain	107	45	62	0.91	81	93	In‐house immunoassay	7

### 3.3 Methodological quality

**FIGURE 2:**
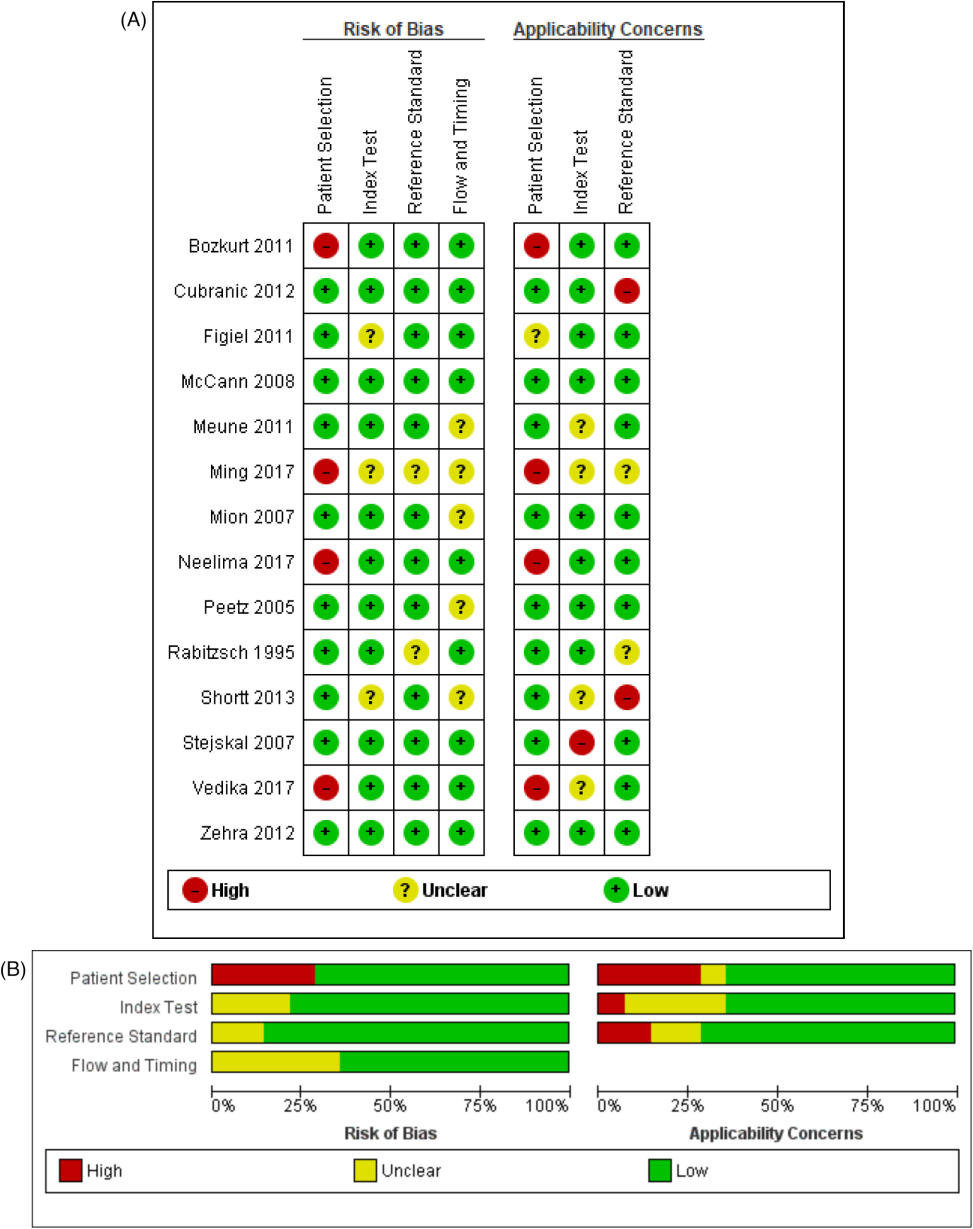
Quality assessments of included studies using the QUADAS‐2 tool. (A) Risk of bias summary: A review of the authors' judgments about the risk of each bias item for each included study. (B) Risk of bias graph: A review of the authors' judgments about each item presented as percentages across all included studies. QUADAS‐2, quality assessment of diagnostic accuracy studies 2

The quality of diagnostic studies was variable as assessed using the QUADAS‐2 tool. The table assessing the risk of bias and concerns regarding the applicability of the GPBB included in the analysis can be accessed in the Appendix S2. The summary and clustered bar graph of studies for risk of bias and applicability are shown in Figure 2.

### 3.4 Meta‐analysis

**FIGURE 3:**
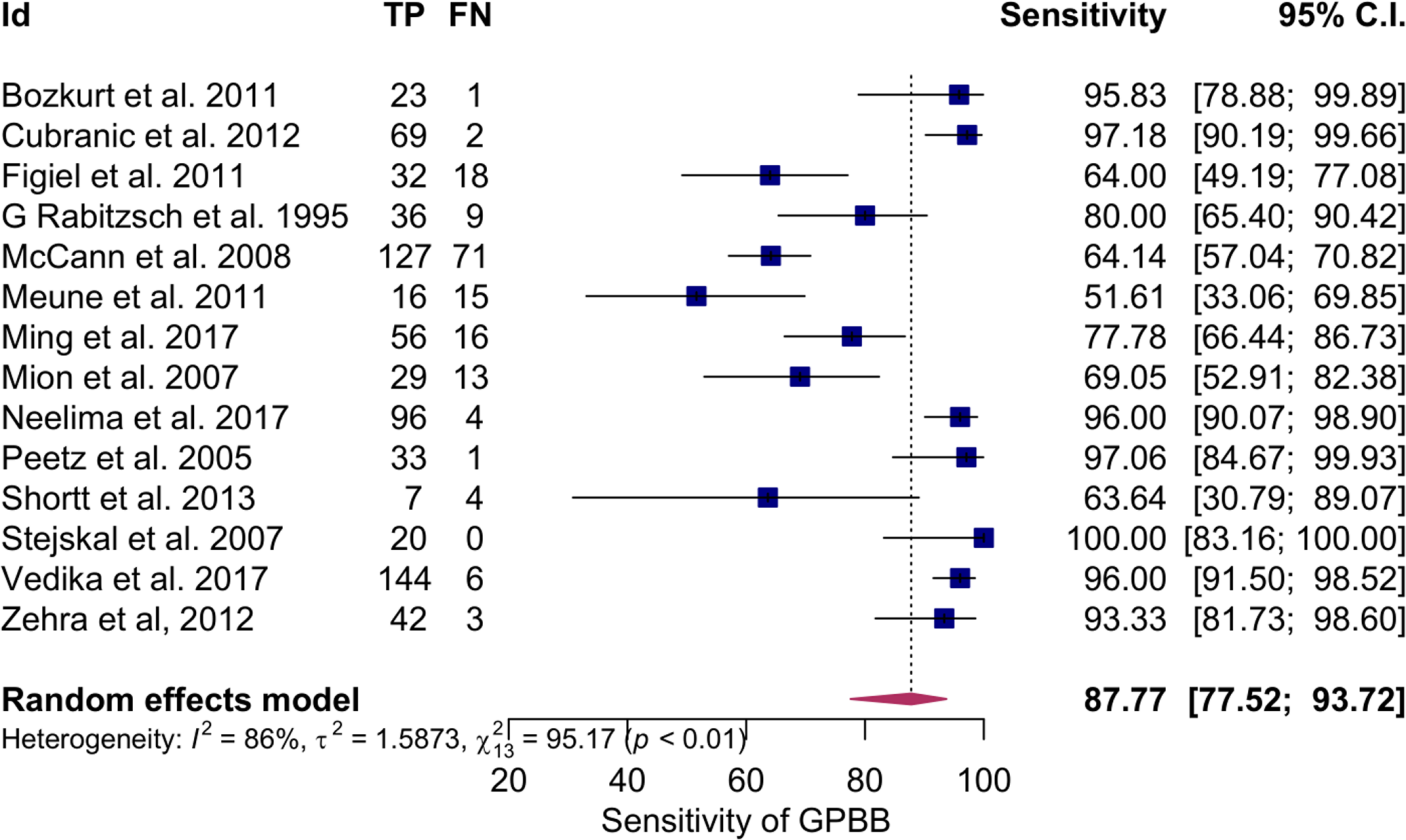
Forest plot showing pooled sensitivity of GPBB for the diagnosis of MI. GPBB, Glycogen Phosphorylase BB; MI, myocardial infarction

**FIGURE 4:**
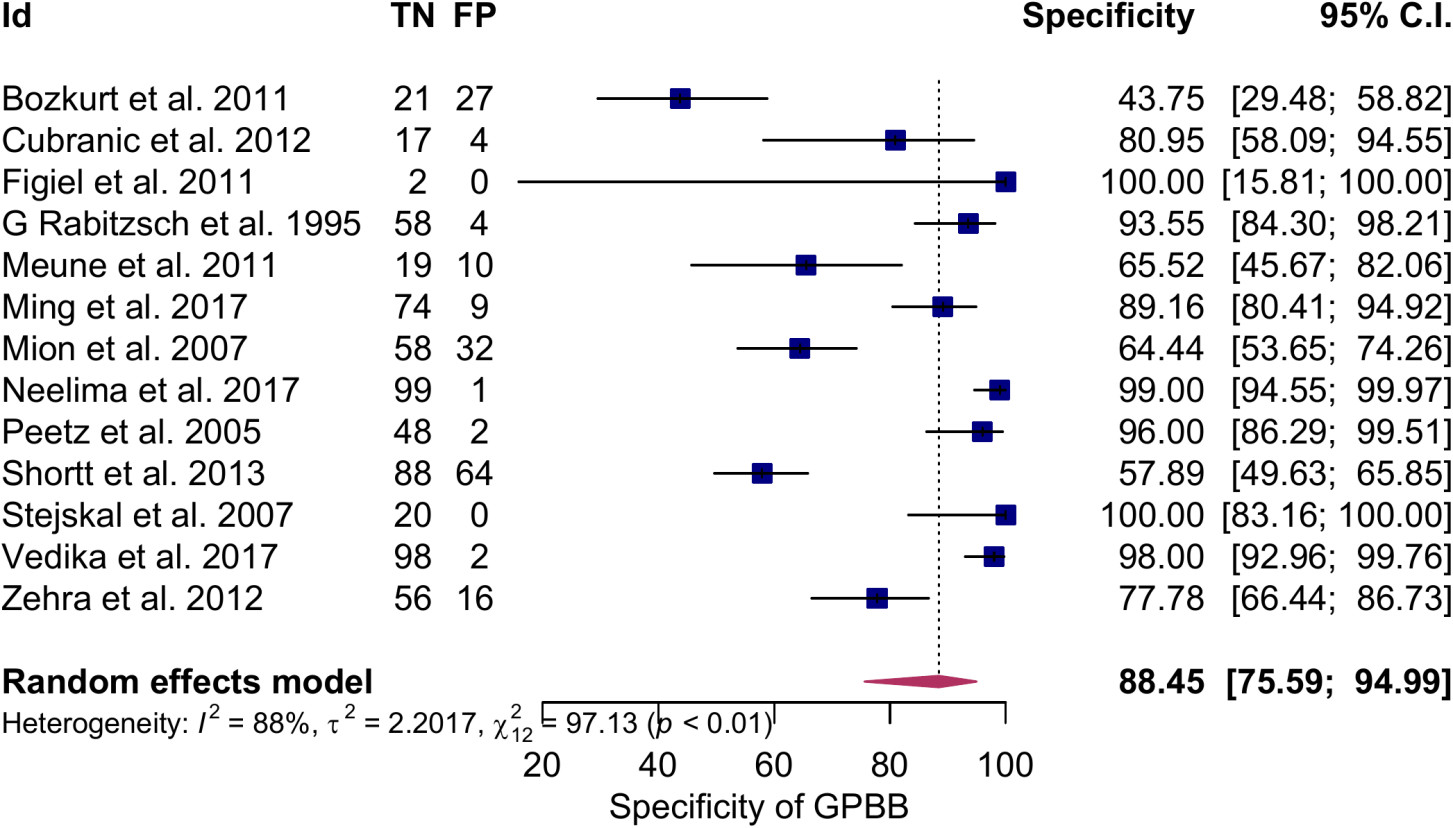
Forest plot showing pooled specificity of GPBB for the diagnosis of MI. GPBB, Glycogen Phosphorylase BB; MI, myocardial infarction

**FIGURE 5:**
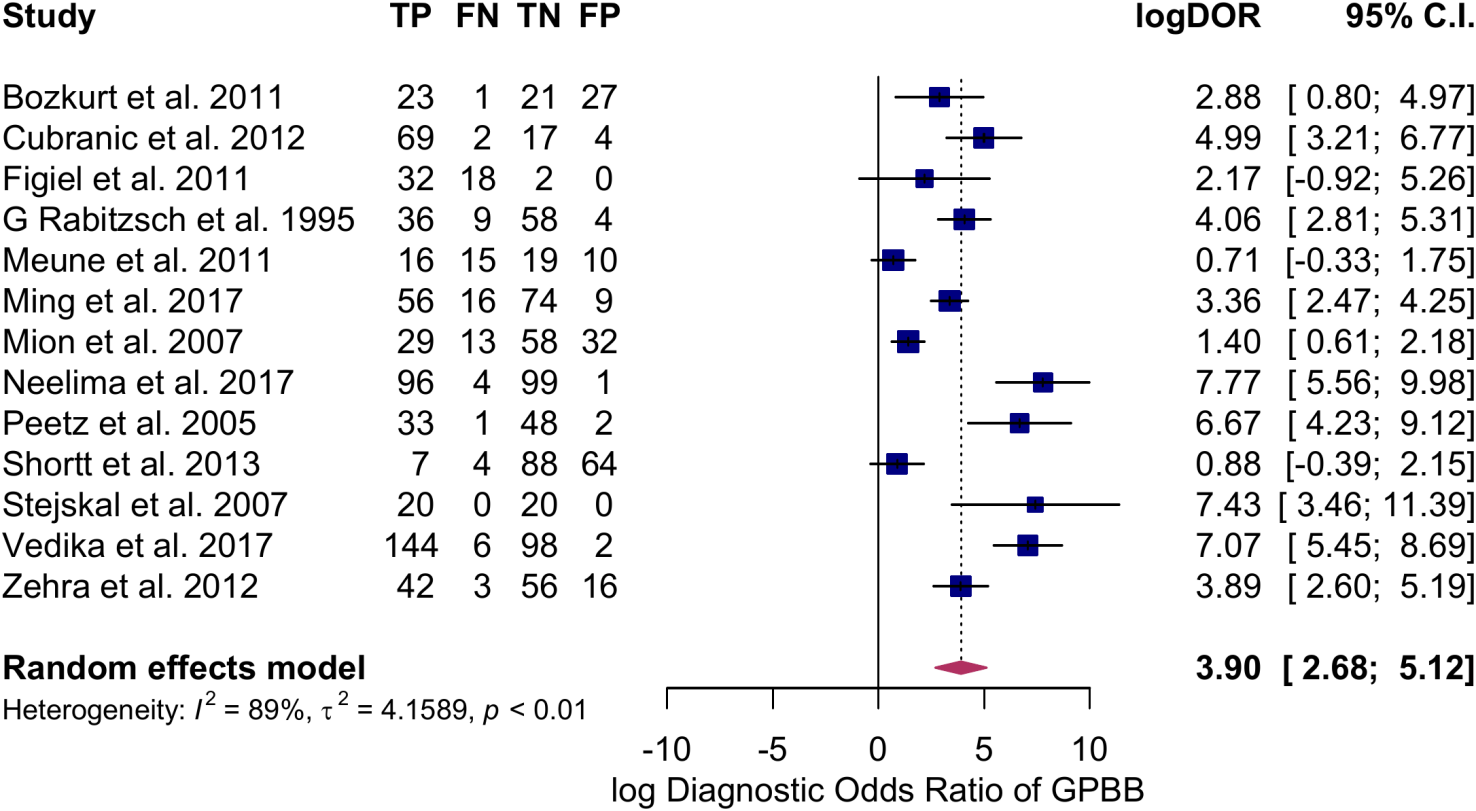
Forest plot showing pooled DOR of GPBB in diagnosing MI. GPBB, Glycogen Phosphorylase BB; MI, myocardial infarction

The summary sensitivity obtained after meta‐analysis was 87.77% (77.52%–93.72%, I^2^ = 86%), summary specificity 88.45% (75.59%–94.99%, I^2^ = 88%), positive likelihood ratio of 5.167 (3.093–8.634, 95% CI), and the negative likelihood ratio of 0.169 (0.096–0.300, 95% CI) and DOR 49.37(14.53–167.72, I^2^ = 89%) using 95% CI. Elevated GPBB was associated with increased MI, log DOR = 3.90 (2.68–5.12, I^2^ = 89%, 95% CI) (Figures 3, 4, 5). There was considerable heterogeneity for all the above‐mentioned statistical measures (I^2^ > 50%), so moderator analysis was conducted for those statistical measures to account for the heterogeneity.

Spearman's rho was calculated to measure the strength of association between sensitivities and the false‐positive rate, which showed a negative correlation between the two variables, rho = −0.432 (95% CI, −0.794–0.156), indicating that the heterogeneity among the included studies could be from other reasons but threshold effect.

### 3.5 HSROC and SROC curve analysis

**FIGURE 6:**
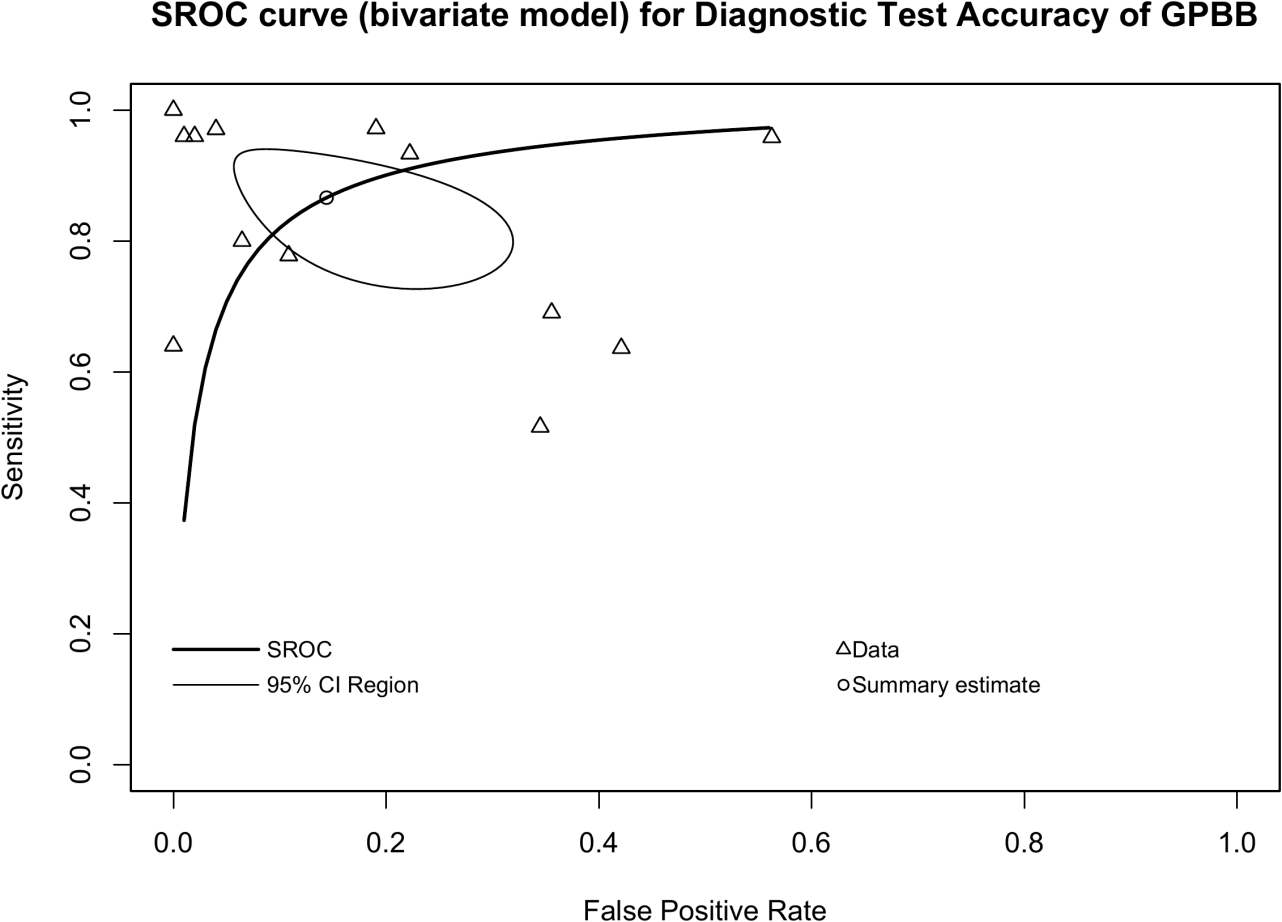
Summary receiver‐operating characteristic curves of the performances of GPBB in the diagnosis of MI. GPBB, Glycogen Phosphorylase BB; MI, myocardial infarction

The sensitivity (*y*‐axis) and false‐positive rate (*x*‐axis) were plotted in a graph to show the relationship between the true positive rate (TPR) and false‐positive rate (FPR) of the test at various thresholds. We used this to distinguish disease cases from noncases (Figure 6). The AUC for SROC was 0.923. The lambda, theta, beta, sigma2alpha, and sigma2theta values of HSROC were 3.670, 0.193, 0.165, 5.378, and 0.267, respectively, where 3.670 estimates the mean of the random effects for accuracy (i.e., lambda), 0.193 estimates the mean of the random effects for threshold (i.e., theta), 0.165 estimates the shape parameter (i.e., beta), 5.378 estimates the variance of the random effects for accuracy (i.e., sigma2alpha), and 0.193 estimates the variance of the random effects for threshold (i.e., sigma2theta). The resulting curve which depicts the expected trade‐off between sensitivity and specificity across thresholds shows that there is certain accuracy of GPBB for diagnosing MI.

### 3.6 Moderator analysis

Considering the heterogeneity obtained on analysis, moderator analysis (subgroup analysis and metaregression) was conducted to explore the effects on heterogeneity and summary estimates. The analysis was conducted by making subgroups under each tentatively homogenous group (sample size, study year, study type, and test kit used). We can see that study design is significantly associated with effect size differences, suggesting that study design (*p* = 0.1816) might be the potential source of heterogeneity. Metaregression using diagnostic odds ratio as the effect size explained that some of the variability in our effect size data may be due to study design (*R* ^2^ = 9.86%). Pooled sensitivity, specificity, and DOR for each subgroup are calculated, and the findings obtained are shown in Table 2.

**TABLE 2:** Moderator analysis (subgroup and metaregression) exploring effects on pooled sensitivity and specificity, pooled DOR, and heterogeneity

Subgroups	Pooled sensitivity (%)	Pooled specificity (%)	Pooled DOR	I^2^ for sensitivity/specificity/DOR (%)	Metaregression *p*‐Value
Sample size (n)					
n < 100	92.65	87.01	51.56	90/89/86	0.979
n > 100	84.80	89.44	50.63	87/95/92	
Study design					
Prospective	0.8876	0.8223	51.065	85/92/57	0.182
Cross‐sectional	0.7669	0.6761	8.807	84/0/83	
Others	0.9163	0.9540	191.945	82/93/92	
Test kit used					
Diacordon	90.28	84.63	48.79	92/89/85	0.970
Others	85.58	90.97	51.90	86/96/93	
Study year					
Before 2010	86.72	92.99	89.60	90/86/90	0.617
After 2010	88.28	85.52	40.58	89/94/90	

### 3.7 Fagan plot

**FIGURE 7:**
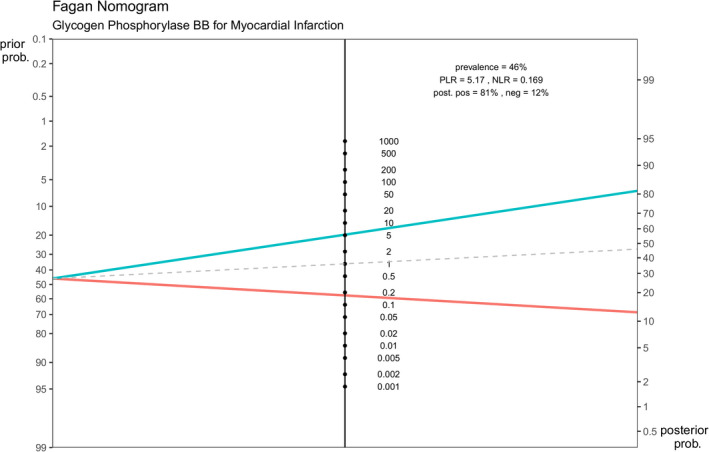
Fagan plot showing the pretest and post‐test probability of GPBB in diagnosing MI. GPBB, Glycogen Phosphorylase BB; MI, myocardial infarction

A Fagan plot was constructed using pretest probability plotted on the vertical axis on the left, likelihood ratio plotted in middle, and post‐test probability plotted on the vertical axis on right. The positive likelihood ratio is 5.167 (3.093–8.634, 95% CI), and the negative likelihood ratio is 0.169 (0.096–0.300, 95% CI). With a pretest probability (prevalence) of MI of 46%, the post‐test probabilities of MI: post‐positive and post‐negative probabilities of MI were found to be 81% and 12% respectively. The post‐positive probability is indicated in the blue line, and the post‐negative probability is indicated in the red line in the Figure 7.

### 3.8 Sensitivity analysis

Sensitivity analysis was done by excluding one study at a time (leave‐one‐out method) that showed no significant differences in the pooled sensitivity, pooled specificity, and DOR (Appendix S3). Pooled sensitivity ranged from 86.25% to 89.26%, pooled specificity from 85.61% to 90.29%, and DOR from 3.5780 to 4.1990.

### 3.9 Publication bias

**FIGURE 8:**
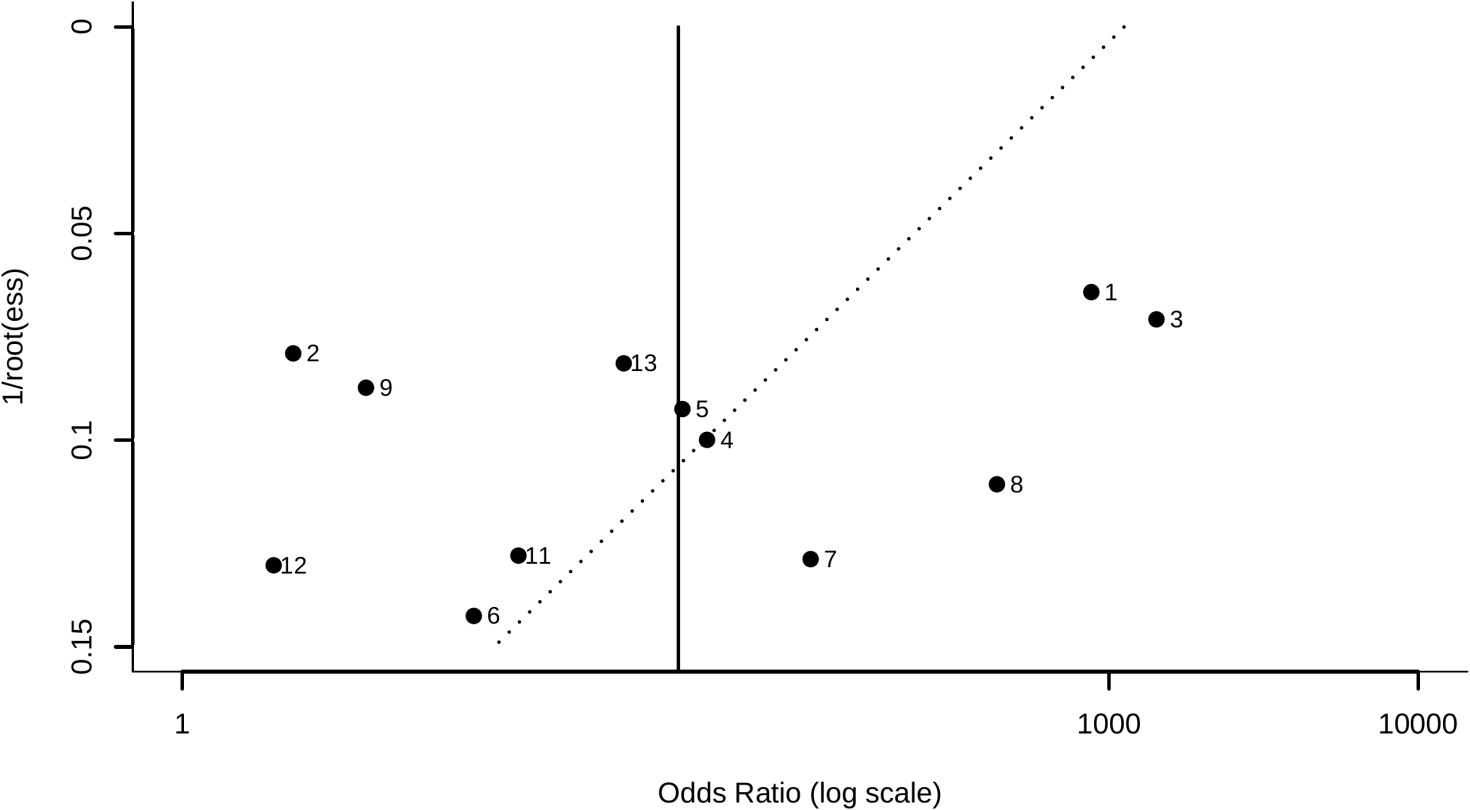
The result of a Deeks' test for assessing the publication bias of GPBB in the diagnosis of MI. GPBB, Glycogen Phosphorylase BB; MI, myocardial infarction

A Deeks' funnel plot was used to assess the publication bias, and it is shown in Figure 8. The figure obtained is symmetrical. The Deeks' funnel plot showed no evidence of publication bias (*p* = 0.1288).

## 4 DISCUSSION

Timely diagnosis and prompt treatment of MI patients significantly decrease MI‐associated morbidity and mortality. However, failing to diagnose the condition early stands as a major hindrance in achieving satisfactory outcomes among MI patients. Despite technological advancements, the diagnosis of MI is still a challenging one and each case poses a unique set of diagnostic challenges. Troponin T and CK‐MB are widely accepted as the standards for MI diagnosis. However, there is still a latency of 7 h before these markers rise to detectable levels.^38^ Moreover, a 10% clinical coefficient is recommended at the 99th percentile for satisfactory clinical confidence in diagnosing MI. The clinical assays currently employed in utilizing cardiac troponins for diagnosis fail to meet this. Though a single time‐point cTn testing may be useful to rule out, such a strategy does not detect the rising and falling pattern required for diagnosis as suggested in the universal definition of AMI. Though there is an increase in the diagnostic sensitivity with a value in the 99th percentile, laboratorians still prefer the 10% coefficient of variation cutoff value.^48^ This has been well‐established for other immunoassay tests, and this margin of safety brings familiarity and comfort. In this context, GPBB has been proposed to be the initial marker to rise in some of the previous studies and a promising tool for the diagnosis of MI.

Biochemical markers have become an integral part of the diagnostic tools for MI. Different reviews have concluded them to be a major domain for preventive, diagnostic, and follow‐up strategies for MI cases.^9^ GPBB is abundant in normal tissues of the brain and myocardium.^39^ GPBB, bound to the sarcoplasmic reticulum glycogenolytic complex of cardiomyocytes, is the key enzyme for glycogenolysis.^40^, ^41^ The myocardial metabolic state determines the level of association between GP with sarcoplasmic reticulum glycogenolytic complex and is found to be highly sensitive to ischemia‐induced glycogenolysis.^41^ Under ischemia during phosphorolysis, the bound form of GPBB becomes a soluble cytosolic form. As under the myocardial ischemia, glycogenolysis is increased and the cell membrane integrity is also lost resulting in GPBB release into extracellular space via the T‐tubule system.^37^, ^42^, ^43^ As the blood‐brain barrier usually remains intact in patients with MI, it is more likely that the increase in the plasma GPBB concentration during chest pain is solely attributable to the GPBB release from the heart.

The role of GPBB as a reliable diagnostic marker for the early diagnosis of MI was proposed first in 1995 when Rabitzsch et al. established GPBB as the most sensitive marker for the diagnosis of AMI within 4 h after the onset of chest pain. The study clearly states that GPBB was the only marker to rise within 2 h of myocardial injury and returned to the baseline soon after.^37^ Since then, a handful of studies have been published, some of which show results in favor of using GPBB for diagnosis, especially in the early hours after MI. Peetz et al. compared GPBB with troponin, myoglobin, and CK‐MB and showed early high sensitivity and specificity of GPBB. Recent studies by Vedika et al. and Neelima et al. showed that GPBB has better sensitivity and specificity in earlier hours (within 4 h of pain onset) than some of the other biomarkers (myoglobin and CK‐MB).^5^, ^25^, ^36^ Early recognition and management are integral for a more favorable prognosis after an acute MI. The use of GPBB for early assessment ensures this. Initial clinical trials suggested a high sensitivity and good specificity of GPBB assays for the early detection of myocardial infarction compared with the other biomarkers, including first‐generation cardiac troponin T.^36^ First generations of the troponin assays had unsatisfactory sensitivity, especially in the early phase of myocardial infarction.^49^ Interestingly, recent data suggest that elevated GPBB may add prognostic information beyond hs‐cTnI and brain natriuretic peptide (BNP). An association between the increased level of GPBB in a patient presenting with symptoms suggestive of ACS and a poorer midterm outcome has also been established.^44^ Furthermore, studies found GPBB to be superior to cTnI and cTnT in a study for the diagnosis of anthracycline‐induced cardiotoxicity and carbon monoxide‐associated cardiotoxicity, both of which pertain to ischemic pathophysiology.^50^, ^51^ Anti‐GPBB antibody clones, which are highly specific and with no cross‐reactivity to GPLL and GPMM, are already developed (but are not yet approved for therapeutic use), which may be suitable for sensitive GPBB assay development. Thus, GPBB may be a simple point‐of‐care test, easy to use, and fast, achieving high diagnostic accuracy. Stejskal et al. 2007^35^ concluded GPPB POCT test can expand the available laboratory diagnostic tools for acute coronary syndrome especially for diagnosis in the first hours, as they observed absolute correspondence between the result of the ELISA test and the qualitative GPBB test in the POCT regime. Similarly, a study using combination testing of the early marker GPBB with CK‐MB and cTnT on the µPAD (microfluidic paper‐based device) reached the conclusion that µPAD POCT would be the best and economic approach to prompt diagnosis during early myocardial ischemia and to prevent an adverse cardiac event.^54^

Studies highlighting GPBB's limitations have also been conducted. HFAB in combination with cardiac troponin at the time of admission has been shown to yield better diagnostic results, especially in the early hour of presentation.^33^ Furthermore, it has recently been demonstrated that GPBB does not improve the diagnostic performance of high‐sensitive troponin I among people with suspected acute coronary syndrome.^52^ A recent study on various biomarkers for MI concluded no single marker (including GPBB) is superior to high‐sensitive cardiac troponins for the diagnosis of acute myocardial infarction.^53^ Similarly, a study by Mion et al. concludes that when GPBB and troponin were used simultaneously for diagnostic purposes, the results were comparable as to troponin used alone. This also raises the question of whether GPBB should be studied with the same level of interest.^34^

The analysis of our pooled data gave results in favor of GPBB as a diagnostically accurate marker, with the log transformation of DOR as 3.9. The result contradicts a previous meta‐analysis done on the same topic. The meta‐analysis done by Lippi et al. in 2013^45^ concluded that GPBB did not meet the requirement for an accurate diagnostic tool. We have incorporated six additional studies and data that report cases within 6 h of symptoms onset in our analysis, one published in 2013 and others after that. This could be one of the factors that led to a different conclusion with our pooled sensitivity and specificity being 87.77% (77.52%–93.72%, I^2^ = 86%) and 88.45% (75.59%–94.99%, I^2^ = 88%), respectively, using 95% CI. The results obtained also show that the diagnostic utility of GPBB for MI is more sensitive compared with the high‐sensitive troponin assay. This is in comparison with a study from 2015 by W.J et al. to outline the diagnostic and prognostic utility of high‐sensitive troponin assays in the early phase (<3 h) of MI, which showed pooled sensitivity and specificity of 0.79 (95% CI 0.71–0.85) and 0.92 (95% CI 0.88–0.96), respectively.^46^ However, the specificity of these assays is higher compared to that of GPBB that may be inferred from a study by Lippi et al., which concluded that even a submaximal aerobic exercise influences the concentration of several markers of muscle damage, one of which is GPBB.^47^ The positive likelihood ratio, negative likelihood ratio, and the AUC for SROC of 5.167, 0.169, and 0.923, respectively, reiterates that GPBB is a sensitive and specific biomarker for the diagnosis of MI.

We conducted further analysis to account for the source of heterogeneity among the included studies. To minimize the heterogeneity, we had set rigorous inclusion/exclusion criteria earlier. The value of I^2^ of pooled summary estimates was >50%, which indicated the existence of heterogeneity in this study. The threshold effect and nonthreshold effects were analyzed to discover the heterogeneous sources. The Spearman's correlation index between sensitivity and false‐positive rate was −0.432 (95% CI, −0.794–0.156), and the ROC plane showed the absence of typical shoulder arm, meaning heterogeneity was not from threshold effects. Moreover, in nonthreshold effect analysis, the result of diagnostic OR (I^2^: 89%) indicated the presence of nonthreshold effect in the included studies. Considering the possible moderators, subgroup and metaregression analyses were conducted to investigate its heterogeneity. The study design was thus found to have some effect on heterogeneity *R* ^2^ = 9.86%, i.e., it accounts for 9.86% of heterogeneity. To address the heterogeneity originating from nonthreshold effect further, sensitivity analysis was conducted by checking the influence of each study. However, no significant change was seen on heterogeneity on the serial omission of the studies, one at a time. Some studies used a defined cutoff value in advance, some used an optimal cutoff by SROC, and several studies did not report cutoff value in the publications. This lack of a definitive range for cutoff values of GPBB, with newer studies using a range from 7 to 19, could be one of the contributing factors to the large heterogeneity.

### 4.1 Limitations of the study

The literature reviewing process was elaborate, but we could only extract studies from limited databases. Studies in a language other than English were not reviewed. There is significant heterogeneity in the study, which could have been a source of bias. We failed to analyze the time gap between sample blood collection to the onset of symptoms due to insufficient data. Our study has not analyzed the adjunctive role of GPBB in combination with other currently used biomarkers for the diagnosis of MI.

## 5 CONCLUSION

With the results of our analysis, we conclude that GPBB had moderate accuracy for diagnosis of MI and was superior to CK‐MB and myoglobin. Whether GPBB assays could potentially replace hsCTn, especially during early hours of symptoms onset remains unclear. For more conclusive evidence and for the GPBB to be made a standard cardiac biomarker for diagnosis in clinical practice, prospective studies employing rigorous laboratory and study design with robust criteria are needed to determine the clinical usefulness of these tests. Keeping the limitations of the study in mind, results may be subject to change in meta‐analyses wherein more data have been extracted from a larger number of studies.

## CONFLICT OF INTEREST

The authors have declared that no competing interests exist.

## AUTHOR CONTRIBUTIONS

AG and SG designed the study. AG, SG, and AB were engaged in the literature search. AT, NK, and SR carried out the title and abstract screening. AG, NK, and SR were involved in full‐text screening. Similarly, data extraction and quality assessment were done by each of the authors independently. AG carried out the statistical analysis, and all authors (AG, SG, NK, SR, AT, AB, and SD) drafted the manuscript. All authors were involved in revising the manuscript critically for important intellectual content. All authors read and approved the final manuscript.

## Supporting information

Appendix S1

Appendix S2

Appendix S3

## Data Availability

The authors confirm that the data supporting the findings of this study are available within the article and its [Link], [Link], [Link].
