# Supplementary material for: Diagnostic accuracy of glycogen phosphorylase BB for myocardial infarction: A systematic review and meta‐analysis
Source: J Clin Lab Anal. 2022 Mar 24;36(5):e24368. doi: 10.1002/jcla.24368 (PMC9102511; doi:10.1002/jcla.24368)
Supplement: Supplementary file 1 — Appendix S1 [file JCLA-36-e24368-s002.docx]

**Supplementary Content**

**Search Date- March 1^st^ 2021**

**Search Strategy**

***Pubmed:***

Details:

(("acute coronary syndrome"[MeSH Terms] OR ("acute"[All Fields] AND "coronary"[All Fields] AND "syndrome"[All Fields]) OR "acute coronary syndrome"[All Fields] OR ("myocardial ischaemia"[All Fields] OR "myocardial ischemia"[MeSH Terms] OR ("myocardial"[All Fields] AND "ischemia"[All Fields]) OR "myocardial ischemia"[All Fields] OR "coronary artery disease"[MeSH Terms] OR ("coronary"[All Fields] AND "artery"[All Fields] AND "disease"[All Fields]) OR "coronary artery disease"[All Fields] OR ("myocardial"[All Fields] AND "ischemia"[All Fields])) OR ("coronary artery disease"[MeSH Terms] OR ("coronary"[All Fields] AND "artery"[All Fields] AND "disease"[All Fields]) OR "coronary artery disease"[All Fields])) AND ("glycogen phosphorylase"[MeSH Terms] OR ("glycogen"[All Fields] AND "phosphorylase"[All Fields]) OR "glycogen phosphorylase"[All Fields] OR ("glycogen phosphorylase, brain form"[MeSH Terms] OR ("glycogen"[All Fields] AND "phosphorylase"[All Fields] AND "brain"[All Fields] AND "form"[All Fields]) OR "brain form glycogen phosphorylase"[All Fields] OR ("glycogen"[All Fields] AND "phosphorylase"[All Fields] AND "brain"[All Fields] AND "form"[All Fields]) OR "glycogen phosphorylase brain form"[All Fields]) OR ("glycogen phosphorylase bb human"[Supplementary Concept] OR "glycogen phosphorylase bb human"[All Fields] OR "glycogen phosphorylase bb human"[All Fields])))


***Embase:***

Session Results

.......................................................

No. Query Results Results Date

#6. #1 AND #4 AND [english]/lim AND [humans]/lim 48 1 Mar 2021

#5. #1 AND #4 70 1 Mar 2021

#4. #2 OR #3 3,241 1 Mar 2021

#3. 'glycogen phosphorylase bb'/exp 33 1 Mar 2021

#2. 'glycogen phosphorylase'/exp 3,230 1 Mar 2021

#1. 'heart infarction'/exp 409,302 1 Mar 2021

....................................................…

***Google Scholar:***

(“glycogen phosphorylase” OR "glycogen phosphorylase BB") AND ("Myocardial Ischemia" OR"Acute Coronary Disease" OR "Coronary Artery Disease")

Also,

(“GPBB” AND “Myocardial Infarction”)
