# Supplementary material for: Diagnostic accuracy of glycogen phosphorylase BB for myocardial infarction: A systematic review and meta‐analysis
Source: J Clin Lab Anal. 2022 Mar 24;36(5):e24368. doi: 10.1002/jcla.24368 (PMC9102511; doi:10.1002/jcla.24368)
Supplement: Supplementary file 2 — Appendix S2 [file JCLA-36-e24368-s003.docx]

**Supplementary Content**

**QUADAS-2 Appraisal**

| Author | Risk of Bias | | | | Applicability concerns | | |
| --- | --- | --- | --- | --- | --- | --- | --- |
|  | Patient Selection | Index Test | Reference Standard | Flow and Timing | Patient Selection | Index Test | Reference Standard |
| Bozkurt et al. 2011 (1) | High | Low | Low | Low | High | Low | Low |
| Cubranic et al. 2012 (2) | Low | Low | Low | Low | Low | Low | High |
| G Rabitzsch et al. 1995 (3) | Low | Low | Unclear | Low | Low | Low | Unclear |
| Meune et al. 2011 (4) | Low | Low | Low | Unclear | Low | Unclear | Low |
| Ming et al. 2017 (5) | High | Unclear | Unclear | Unclear | High | Unclear | Unclear |
| Mion et al. 2007 (6) | Low | Low | Low | Unclear | Low | Low | Low |
| Neelima et al. 2017 (7) | High | Low | Low | Low | High | Low | Low |
| Peetz et al. 2005 (8) | Low | Low | Low | Unclear | Low | Low | Low |
| Shortt et al. 2013 (9) | Low | Unclear | Low | Unclear | Low | Unclear | High |
| Stejskal et al. 2007 (10) | Low | Low | Low | Low | Low | High | Low |
| Vedika et al. 2017 (11) | High | Low | Low | Low | High | Unclear | Low |
| Zehra et al. 2012 (12) | Low | Low | Low | Low | Low | Low | Low |
| Figiel et al. 2011 (13) | Low | Unclear | Low | Low | Unclear | Low | Low |
| McCann et al. 2008 (14) | Low | Low | Low | Low | Low | Low | Low |

***References***

1. Bozkurt S, Kaya EB, Okutucu S, Aytemir K, Coskun F, Oto A. The diagnostic and prognostic value of first hour glycogen phosphorylase isoenzyme BB level in acute coronary syndrome. Cardiol J [Internet]. 2011 [cited 2021 May 20];18(5):496–502. Available from: https://pubmed.ncbi.nlm.nih.gov/21947984/

2. Cubranic Z, Madzar Z, Matijevic S, Dvornik S, Fisic E, Tomulic V, et al. Diagnostic accuracy of heart fatty acid binding protein (H-FABP) and glycogen phosphorylase isoenzyme BB (GPBB) in diagnosis of acute myocardial infarction in patients with acute coronary syndrome. Biochem Medica [Internet]. 2012 [cited 2021 May 20];22(2):225–36. Available from: /pmc/articles/PMC4062338/

3. Rabitzsch. Immunoenzymometric assay of human glycogen phosphorylase isoenzyme BB in diagnosis of ischemic myocardial injury - PubMed [Internet]. [cited 2021 May 20]. Available from: https://pubmed.ncbi.nlm.nih.gov/7600699/

4. Meune C, Wahbi K, Weber S, Zuily S, Cynober L, Chenevier-Gobeaux C. Performance of glycogen phosphorylase isoenzyme BB is weak in the detection of patients with non-ST-elevation acute coronary syndrome. Clin Biochem [Internet]. 2011 Nov [cited 2021 May 20];44(16):1343–5. Available from: https://pubmed.ncbi.nlm.nih.gov/21820423/

5. Ming. Evaluation of clinical performance of glycogen phosphorylase BB in diagnosis and prognosis of non-ST elevation acute coronary syndrome [Internet]. [cited 2021 May 20]. Available from: http://journal08.magtechjournal.com/Jwk_wjyx/EN/abstract/abstract15811.shtml#

6. Mion MM, Novello E, Altinier S, Rocco S, Zaninotto M, Plebani M. Analytical and clinical performance of a fully automated cardiac multi-markers strategy based on protein biochip microarray technology. Clin Biochem. 2007 Nov 1;40(16–17):1245–51.

7. Singh N, Rathore V, Mahat RK, Rastogi P. Glycogen Phosphorylase BB: A more Sensitive and Specific Marker than Other Cardiac Markers for Early Diagnosis of Acute Myocardial Infarction. Indian J Clin Biochem [Internet]. 2018 Jul 1 [cited 2021 May 20];33(3):356–60. Available from: https://link.springer.com/article/10.1007/s12291-017-0685-y

8. Peetz D, Schweigert R, Schollmayer C, Steinbach K, Lackner KJ, Post F, et al. Glycogen Phosphorylase BB in Acute Coronary Syndromes. Clin Chem Lab Med [Internet]. 2005 [cited 2021 May 20];43(12):1351–8. Available from: https://pubmed.ncbi.nlm.nih.gov/16309372/

9. Shortt CR, Worster A, Hill SA, Kavsak PA. Comparison of hs-cTnI, hs-cTnT, hFABP and GPBB for identifying early adverse cardiac events in patients presenting within six hours of chest pain-onset. Vol. 419, Clinica Chimica Acta. Elsevier; 2013. p. 39–41.

10. Stejskal D, Lacnak B, Jedelsky L, Stepanova L, Proskova J, Solichova P, et al. Use of glycogen phosphorylase BB measurement with POCT in the diagnosis of acute coronary syndromes. A comparison with the ELISA method. Biomed Pap Med Fac Univ Palacky Olomouc Czech Repub [Internet]. 2007 [cited 2021 May 20];151(2):247–9. Available from: https://pubmed.ncbi.nlm.nih.gov/18345258/

11. Rathore V, Rastogi P, S CY, Singh N, Kumar Mahat R. Comparative status of glycogen phosphorylase BB, myoglobin, and CK-MB for early diagnosis of acute myocardial infarction. Int J Med Sci [Internet]. 2017 [cited 2021 May 20]; Available from: http://www.ijmsph.com

12. Zehra. GLYCOGEN PHOSPHORYLASE ISOENZYME BB IN EARLY DIAGNOSIS OF ACUTE CORONARY SYNDROME [Internet]. [cited 2021 May 20]. Available from: https://www.nobelmedicus.com/en/Article.aspx?m=337

13. Figiel. Direct comparison of the diagnostic value of point-of-care tests detecting heart-type fatty acid binding protein or glycogen phosphorylase isoenzyme BB in patients with acute coronary syndromes with persistent ST-segment elevation - PubMed [Internet]. [cited 2021 May 20]. Available from: https://pubmed.ncbi.nlm.nih.gov/21267954/

14. McCann CJ, Glover BM, Menown IBA, Moore MJ, McEneny J, Owens CG, et al. Novel biomarkers in early diagnosis of acute myocardial infarction compared with cardiac troponin T. Eur Heart J [Internet]. 2008 Dec [cited 2021 May 20];29(23):2843–50. Available from: https://pubmed.ncbi.nlm.nih.gov/18682444/
