# Supplementary material for: Diagnostic accuracy of glycogen phosphorylase BB for myocardial infarction: A systematic review and meta‐analysis
Source: J Clin Lab Anal. 2022 Mar 24;36(5):e24368. doi: 10.1002/jcla.24368 (PMC9102511; doi:10.1002/jcla.24368)
Supplement: Supplementary file 3 — Appendix S3 [file JCLA-36-e24368-s001.docx]

**Supplementary Content**

**Sensitivity Analysis (Leave-one-out)**

***Sensitivity analysis of Specificity***

| Author | Specificity | tau^2^ | I^2^ |
| --- | --- | --- | --- |
| Omitting Bozkurt et al. 2011 | 0.9029 [0.7924; 0.9578] | 1.8809 | 86.50% |
| Omitting Cubranic et al. 2012 | 0.8912 [0.7516; 0.9569] | 2.461 | 88.50% |
| Omitting Figiel et al. 2011 | 0.8796 [0.7457; 0.9479] | 2.2191 | 88.70% |
| Omitting G Rabitzsch et al. 1995 | 0.8792 [0.8541; 0.9005] | 2.373 | 86.90% |
| Omitting Meune et al. 2011 | 0.8981 [0.7692; 0.9588] | 2.3104 | 88.60% |
| Omitting Ming et al. 2017 | 0.8860 [0.7402; 0.9549] | 2.4842 | 86.80% |
| Omitting Mion et al. 2007 | 0.8990 [0.7718; 0.9591] | 2.2799 | 88.50% |
| Omitting Neelima et al. 2017 | 0.8561 [0.7208; 0.9320] | 1.703 | 86.80% |
| Omitting Peetz et al. 2005 | 0.8732 [0.7280; 0.9466] | 2.2187 | 87.30% |
| Omitting Shortt et al. 2013 | 0.9008 [0.7790; 0.9590] | 2.1613 | 86.60% |
| Omitting Stejskal et al. 2007 | 0.8655 [0.7293; 0.9389] | 1.9534 | 88.70% |
| Omitting Vedika et al. 2017 | 0.8633 [0.7216; 0.9390] | 1.9386 | 86.00% |
| Omitting Zehra et al. 2012 | 0.8941 [0.7567; 0.9582] | 2.4545 | 88.40% |

***Sensitivity analysis of Sensitivity***

| Author | Sensitivity | tau^2^ | I^2^ |
| --- | --- | --- | --- |
| Omitting Bozkurt et al. 2011 | 0.8686 [0.7573; 0.9334] | 1.5838 | 86.80% |
| Omitting Cubranic et al. 2012 | 0.8625 [0.7515; 0.9287] | 1.4557 | 85.50% |
| Omitting Figiel et al. 2011 | 0.8902 [0.7902; 0.9458] | 1.5883 | 87.00% |
| Omitting G Rabitzsch et al. 1995 | 0.8841 [0.7743; 0.9443] | 1.7545 | 87.30% |
| Omitting McCann et al. 2008 | 0.8907 [0.8696; 0.9087] | 1.5758 | 84.50% |
| Omitting Meune et al. 2011 | 0.8926 [0.8003; 0.9452] | 1.4133 | 86.20% |
| Omitting Ming et al. 2017 | 0.8855 [0.7770; 0.9449] | 1.7412 | 87.30% |
| Omitting Mion et al. 2007 | 0.8885 [0.7852; 0.9456] | 1.6553 | 87.30% |
| Omitting Neelima et al. 2017 | 0.8657 [0.7520; 0.9320] | 1.5461 | 84.50% |
| Omitting Peetz et al. 2005 | 0.8653 [0.7547; 0.9306] | 1.5123 | 86.60% |
| Omitting Shortt et al. 2013 | 0.8882 [0.7861; 0.9450] | 1.627 | 87.30% |
| Omitting Stejskal et al. 2007 | 0.8623 [0.7555; 0.9270] | 1.4171 | 87.40% |
| Omitting Vedika et al. 2017 | 0.8653 [0.7510; 0.9318] | 1.542 | 82.30% |
| Omitting Zehra et al, 2012 | 0.8720 [0.7578; 0.9368] | 1.6732 | 86.40% |

***Sensitivity analysis of DOR***

| Author | logDOR | tau^2^ | I^2^ |
| --- | --- | --- | --- |
| Omitting Bozkurt et al. 2011 | 3.9882 [2.6892; 5.2871] | 4.3797 | 90.10% |
| Omitting Cubranic et al. 2012 | 3.8089 [2.5284; 5.0894] | 4.2108 | 89.60% |
| Omitting Figiel et al. 2011 | 4.0115 [2.7365; 5.2865] | 4.2711 | 90.10% |
| Omitting G Rabitzsch et al. 1995 | 3.8981 [2.5703; 5.2259] | 4.5481 | 89.80% |
| Omitting Meune et al. 2011 | 4.1990 [2.9476; 5.4505] | 3.9356 | 87.90% |
| Omitting Ming et al. 2017 | 3.9827 [2.5806; 5.3849] | 5.1477 | 90.00% |
| Omitting Mion et al. 2007 | 4.1543 [2.8414; 5.4671] | 4.4015 | 88.10% |
| Omitting Neelima et al. 2017 | 3.5780 [2.3922; 4.7638] | 3.5364 | 88.10% |
| Omitting Peetz et al. 2005 | 3.6888 [2.4515; 4.9261] | 3.9327 | 89.20% |
| Omitting Shortt et al. 2013 | 4.1805 [2.9001; 5.4609] | 4.1729 | 89.00% |
| Omitting Stejskal et al. 2007 | 3.7220 [2.4847; 4.9594] | 4.0393 | 89.70% |
| Omitting Vedika et al. 2017 | 3.5834 [2.4223; 4.7445] | 3.3164 | 87.10% |
| Omitting Zehra et al. 2012 | 3.9143 [2.5848; 5.2438] | 4.5653 | 89.90% |
